# *“They come and knock at the gate until the neighbours see”*. Perceived barriers and benefits of implementing HIV care at the community level in Tshwane district: A qualitative study

**DOI:** 10.1371/journal.pone.0240740

**Published:** 2020-10-15

**Authors:** Sanele Ngcobo, Theresa Rossouw

**Affiliations:** 1 Department of Family Medicine, University of Pretoria, Pretoria, South Africa; 2 Department of Immunology, University of Pretoria, Pretoria, South Africa; Federal University of Sergipe, BRAZIL

## Abstract

Little is known about the barriers and benefits of home-based HIV services offered by community health workers. These are especially important as the South African government embarks on scaling up community-based health services, which include HIV care. This study set out to understand potential benefits and barriers of these services in Tshwane district and develop recommendations for improvement. From June to August 2019, seven focus group discussions were conducted with 58 participants: four with 36 ward-based outreach team (WBOT) members and three with 22 people living with HIV (PLWHIV). Three aspects of care were explored: 1. Experience of performing, receiving or observing home-based HIV care; 2. Barriers to conducting home visits; and 3. The perceived value of WBOTs and home-based HIV care. While home-based HIV care was seen as a support strategy which could motivate patients to take their medication, the unpredictability of patients’ responses to HIV test results, incorrect addresses (driven by the need for identity documents), fear of stigma through association with WBOTs, especially those in uniform, little or no preparation of patients for home-based care, and lack of confidentiality and trust were raised as potential barriers. To successfully implement effective home-based HIV care in South Africa, perceived barriers should be addressed and recommendations offered by people providing and receiving these services should be seriously considered. Pertinent recommendations include integrating WBOTs into clinics and existing support structures, improving training on confidentiality and HIV testing, and rethinking the recruitment, scope of work and safety of WBOTs. In addition, research should be conducted into the impact of the requirements for identity documents and community health worker uniforms.

## 2 Introduction

South Africa has the largest HIV epidemic in the world, with an estimated 7.7 million people living with HIV (PLWHIV) in 2018 [1]. South Africa also has the largest antiretroviral treatment (ART) programme globally, with an estimated 4.8 million people on ART [2]. Optimal adherence to ART in persons with HIV improves health outcomes and prevents drug resistance [3]. A systematic review by Mills and colleagues obtained a pooled estimate of adequate adherence in sub-Saharan African patients of 77% (95% confidence interval 68–85%) based on a total of 12,116 patients [4]. Traditionally, sub-optimal adherence is defined as <95%, although it appears that current ART regimens require 70–90% adherence in order to be effective [5]. In addition to sub-optimal adherence, large treatment programmes are plagued by high numbers of patients who become lost to care.

Barriers to HIV care at the facility level are well documented in the literature. Amongst the leading barriers are personal, social, financial, geographic/transportation, and health system barriers [6]. New models of health care delivery are needed to improve treatment adherence and patient retention. Community-oriented Primary Care, which makes use of community health workers, has been successful in countries such as Brazil and Spain and is currently piloted in Tshwane district. Community health workers have been reported to improve adherence to ART, enhance the reach, uptake and quality of HIV services, as well as the dignity, quality of life and retention in care of PLWHIV [7]. Their role in the South African HIV response is, however, understudied.

Community health workers are community members who serve as frontline health care workers. They are indigenous—ethnically, linguistically, socioeconomically, and experientially—to the community in which they work [8]. Supervised by a professional nurse or enrolled nurse, they work with the underserved and offer integrated and comprehensive health care services, which include health promotion, disease prevention and early detection, ante- and post-natal care as well as psychosocial support, at community, household and individual level [9]. Community health workers are organised into ward-based outreach teams (WBOTs), which are supervised by an operational team leader, usually a nurse. These health workers are collectively known as WBOT members. They visit patients who are receiving chronic medication at least once a month. In South Africa, community health workers receive a 10-day theory course, followed by practical and in-service training [9,10] which focuses on communication skills, disease prevention, health promotion, screening for different conditions, palliative care and treatment support for chronic conditions [11]. The South African government is currently phasing in a matric requirement for all community health workers.

While the scope of work of community health workers is comprehensive, their focus in the South African setting has been on supportive HIV care. Naidoo [12] summarized the potential contribution of community health workers in this domain according to four themes: prevention of HIV transmission through health education; identification and testing of those at risk; adherence support; and early identification of individuals with deteriorating health while on ART. All these activities should happen at a household level in the community. While this model appears to overcome some of the limitations of facility-based care, little is known about the benefits and barriers of home-based HIV care services offered by community health workers. As the South African government has embarked on scaling up community-based health services, which include HIV care, it is essential to develop a more in-depth understanding of this care modality and explore options to address perceived barriers.

## 3 Methods

### 3.1 Overview

This is a qualitative study that was conducted in eleven clinics across Tshwane district, Gauteng, South Africa, between June and August 2019. Tshwane district covers 6 298 km^2^, has an estimated population of 3,275,152 and has a total of eight community health centres and 68 Clinics. We conducted seven focus group discussions in order to obtain a detailed group perspective about home-based HIV care, perceptions of the benefits and barriers of delivering HIV care in the community by WBOTs and the experience of receiving HIV care by PLWHIV. A focus group discussion methodology was specifically selected since it allows people to discuss everyday situations in a non-threatening environment which encourages disclosure of real attitudes and beliefs around sensitive issues [13].

Ethics approval was granted by the University of Pretoria, Faculty of Health Science’s Research Ethics Committee (reference number: 580/2018). Permission to collect data was obtained from Tshwane health district and all facilities involved. Each participant gave informed consent before participating in the study. No personal identifying information was captured.

### 3.2 Participants and procedures

Three groups of PLWHIV (PLWHIV_1 to 3) and four groups of WBOT members (WBOT 1 to 4) were purposively selected based on their respective experience of living with HIV (PLWHIV) and with home-based HIV care (WBOTs and PLWHIV). This number was deemed adequate to achieve code saturation [14].

PLWHIV_1 was made up of patients who were already receiving home-based HIV care. PLWHIV_2 and PLWHIV_3 were receiving HIV care at a clinic and, while they had not received HIV care in the community, they had observed community health workers in their communities. PLWHIV were invited by health care workers to join the study, either during their home (PLWHIV_1) or clinic (PLWHIV_2 and 3) visit.

**Fig 1:**
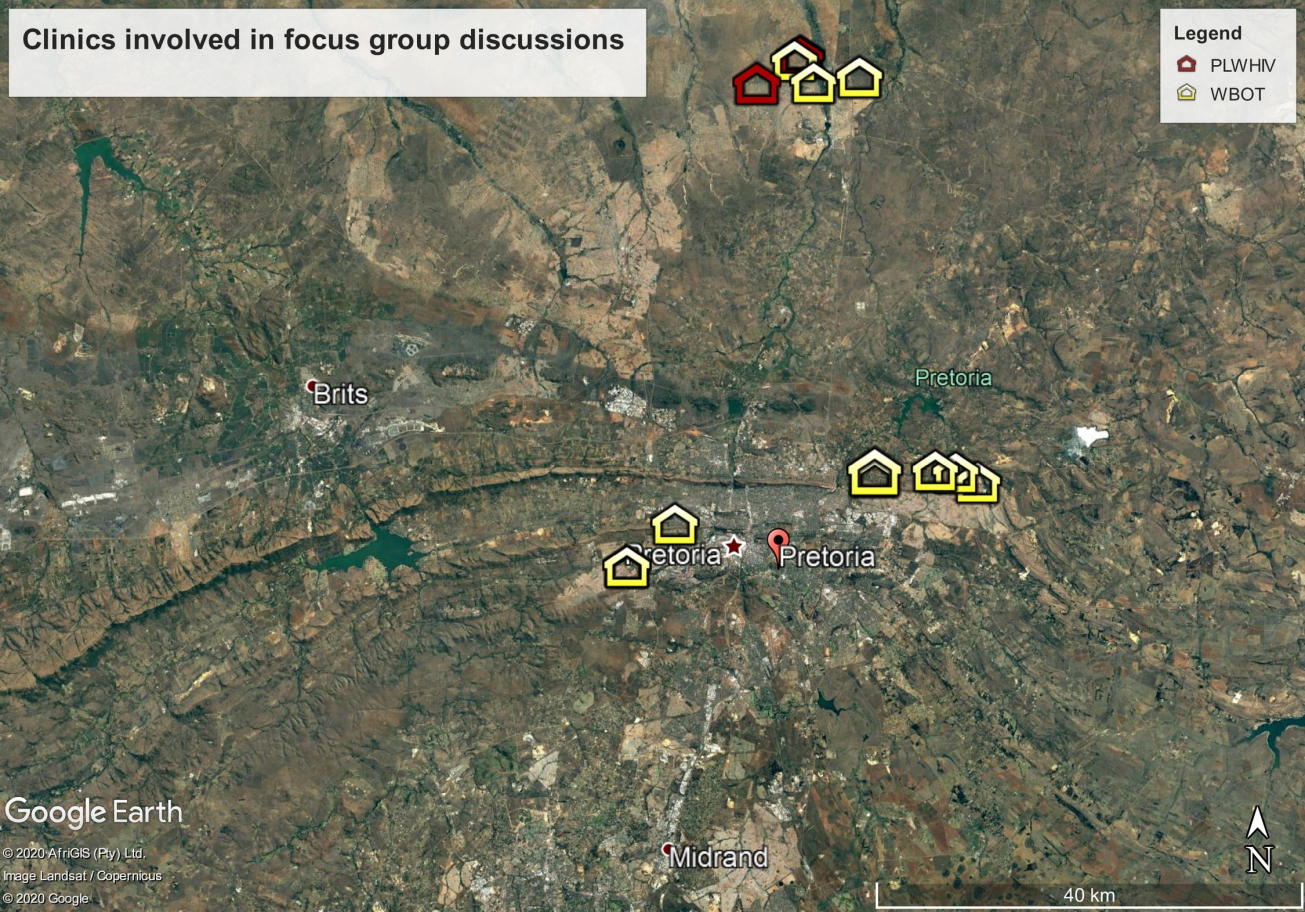
Number of patients with no home address or cell phone number recorded for five clinics on tier.net.

WBOT_1 to WBOT_4 were made up of operational team leaders and community health workers who were delivering HIV care at the community level across Tshwane (Fig 1). In order to capture a diversity of views, WBOTs working in different locations within Tshwane were recruited. WBOTM_2 and 3 covered one clinic each, while WBOT_1 and WBOT_4 covered three and four clinics respectively.

WBOTs who had performed home-based HIV care for at least one year were invited to participate. A focus group discussion guide (S1 Table) was developed by the principal author based on the literature reviewed. The guide was pilot tested in Kekanastad clinic with a group of five community health workers and small changes were made to improve understanding.

With participants’ permission, focus group discussions were audio-recorded and transcribed by a research assistant. Questions centred around three aspects of care 1) Experience of CHWs offering home-based HIV care or experience of PLWHIV receiving home-based HIV care; 2) Barriers to conducting home visits; and 3) Perceived value of WBOTs and home-based HIV care. At the end of the interviews, participants were questioned about a fourth aspect, namely recommendations to improve HIV care in the community.

### 3.3 Data analysis

Data analysis was conducted qualitatively by means of thematic analysis [15]. Castleberry’s thematic approach was used with five different steps: compiling data; dissembling data to different codes; reassembling data to themes; interpretation of themes; and conclusion [15]. The transcripts were read by the principal author multiple times to become familiar with the data, emerging themes and codes. The principal author also discussed his understanding of different themes emerging from the interviews with the transcriber. ATLAS.ti 8 windows was used to arrange, resemble and manage qualitative data, and to facilitate text searching and coding. Codes, concepts and ideas that had some connection with each other were grouped together to form different themes. The quality and boundaries of themes were assessed, followed by analysis of the thickness and thinness of different themes [15]. Diversity and coherence of data were also assessed. Participants’ biographic information was analysed by performing descriptive statistics in SPSS version 26 (IBM Corp., 2019).

## 4 Results

A total of 58 participants were interviewed, as outlined in Table 1. Results of the focus group discussions are presented according to the following four areas of enquiry: 1. Experience of performing, receiving or observing home-based HIV care; 2. Barriers to conducting home visits; 3. The perceived value of WBOTs and home-based HIV care; and 4. Recommendations by participants.

**Table 1:** Description of participants involved in the focus group discussions.

Group	N	Females n (%)	Community health worker (Operational team leader) N (n)	Number of clinics covered	Age (years) Mean (SD)
PLWHIV_1	4	3 (75%)		1	43.5 (16.4)
PLWHIV_2	9	6 (66%)		1*	40.0 (9.5)
PLWHIV_3	9	8 (89%)		1*	44.8 (10.1)
WBOT_1	10	8 (80%)	7 (3)	3	36.0 (1.2)
WBOT_2	7	5 (71%)	6 (1)	1	43.1 (11.4)
WBOT_3	7	7 (100%)	6 (1)	1	32.8 (4.0)
WBOT_4	12	10 (83%)	6 (6)	4	36.1 (6.6)
Total	58	47 (81%)	25 (11)	11	

*PLWHIV 2 and 3 were from the same clinic.

PLWHIV = people living with HIV; n = number; SD = standard deviation; WBOT = ward-based outreach team.

### 4.1 Experience of performing, receiving or observing home-based HIV care

The majority of PLWHIV who have received home-based HIV care were generally satisfied with and those who had observed WBOTs in the community were supportive of the service. Men were especially enthusiastic about the WBOT services and believed that male patients should be prioritised for this service.

“*You see door to door [WBOT services] is good because some of us are afraid to come to the clinic as most man [men]*, *they don’t like coming to the clinic*.*” PLWHIV_2*

Participants providing care, however, reported that it was difficult for them to do HIV counselling and testing at the household level, due to the unpredictability of individuals’ responses.

“*I requested a small table and then sterilised [disinfected] it*, *then explained the process and then allowed the client to sign the consent form*, *then started with pre-testing counselling*. *I then tested the patient*. *When I showed him the results*, *he jumped from where he was sitting straight to the next room*. *By this time*, *I was confused and scared to what is happening to the person in the room but then*, *after some time*, *he came out and we continued with post counselling*.” *WBOT_4*

Other members of the WBOTs believed that performing HIV counselling and testing in the household compromises their safety.

“*It might happen that the person might vent on you after the results*. *It is not a safe place to test; the best place for testing is in the facility*.*” WBOT_4*

WBOTs have to trace patients who have defaulted from tuberculosis (TB) and HIV treatment and this raises the concern that they are at high risk of being infected with these pathogens.

“A*t times during tracing*, *you find that you don’t have a mask and the patient is very sick and you cannot turn back without assisting that client*. *And sometimes you find that they didn’t even open the window*, *so you need to go inside and assist such a client*. *We even have a community health worker who passed away from TB which she contracted while tracing a TB client*.*” WBOT_2*

### 4.2 Barriers to conducting home visits

Despite the perceived value of WBOTs and home-based HIV care, all participants reported perceived barriers to the successful implementation of HIV care at the household level. These barriers were divided into three themes: stigma-related; disclosure and trust; and facility- and health system-related.

#### 4.2.1 Stigma-related barriers

Several stigma-related barriers were raised by both WBOTs and PLWHIV. The most common barriers were around drawing unwelcome attention and the perception that WBOTs only visit people who are ill.

HIV care at the community level involves regular visits to households of PLWHIV. This practice was viewed by some PLWHIV as drawing unwelcome attention.

“*I wouldn’t want them to come and visit me*. *After knowing that you are HIV positive*, *they then visit you every day*, *and they come and knock at the gate until the neighbours see*.*” PLWHIV_2*

PLWHIV felt that the official uniforms of community health workers and Department of Health branding added to the problem.

“*When they first came with their uniform*, *everyone was looking*. *They also had branded cars; it attracted unnecessary attention*.*” PLWHIV_1*

“*Everyone can see who they are*, *what they are doing*. *I requested them to stop wearing uniform when they are coming to see me*.*” PLWHIV_1*

However, wearing a uniform was seen as potentially important by the WBOTs since this facilitated access to some households.

“A *certain woman told us that when we enter a house [without a uniform] they think we are from a religious community*, *so they lock themselves in their houses and watch us through the windows*.” *WBOT_2*

PLWHIV felt that WBOTs are seen as people who only visit people who are ill, thereby potentially adding to stigma.

“*Some people know in the community that those people are only visiting sick patients*.*” PLWHIV_1*

In addition, some PLWHIV indicated that when WBOTMs spend more time at a particular household, it raises suspicion among other community members.

“*When they come and spend too much time at your house*, *people start thinking that someone is sick*.*” PLWHIV_3*

#### 4.2.2 Disclosure and trust

Both WBOTs and community health workers voiced reasons why PLWHIV are reluctant to disclose their status to WBOTs. Community health workers can only know the HIV status of individuals once they disclose to them or if they are provided with a list of those who are HIV-infected by the clinic.

Many PLWHIV were not comfortable disclosing their status to community health workers.

“*The first time they came to me I hide [hid] my identity from them*.” PLWHIV_2

“*When they come to us*, *they are harsh towards us*, *so they must try to being [be] nice and I can disclose to [them]*, *though I don’t think my status will be safe with them*. *It is better if I come to the clinic” PLWHIV_2*

One of the reasons given for not disclosing to CHWs was the fear of gossip.

“*They should not come to me because if there is one who is my neighbour*, *[they] will go around speaking about my health status so and I don’t like that because you end up being stigmatised and be stressed about such gossip*.” PLWHIV_2

Such fears appeared to be grounded in experience. WBOTs reported that there had been a complaint about a CHW who had disclosed individuals’ status to family members without their consent.

“*We once had patients who complained that their status was disclosed to their family without their approval*.*” WBOT_4*

There was also a concern that there might be no benefit of disclosing their status to CHWs.

“*Let’s say I disclose with such a community health worker having little knowledge; what will happen*?*” PLWHIV_2*

While some participants indicated that they would disclose their status to WBOTs, concerns were raised about WBOTs coming from the local area.

“*I can trust them*, *and even disclose my status to them*, *as long as they aren’t staying closer to where I am staying*.*” PLWHIV_ 2*

Having community health workers as neighbours was seen as a barrier which could erode confidentiality and trust.

“*Neighbour never loved their neighbour*. *When they see you*, *they greet you well; but when they get to their home*, *you become a topic*.*” PLWHIV_2*

There was a feeling that community health workers recruited from other areas are only there to do their work and not to judge. Asked if they would trust those coming from other areas, most PLWHIV indicated that they could disclose to them.

“*Yes*, *because they don’t know anything about me and they come there to do their jobs*. *Then that’s fine; I can disclose to such a person unlike the person from the same area*.*” PLWHIV_2*

“*Yes I will disclose*, *and will also be open to them (WBOTM from other areas)*.*” PLWHIV_3*

This sentiment was further supported by the operational team leaders:

“*They don’t trust community health workers that they know*, *that are staying in their community*, *because they think they will disclose their status to everybody in their community; as a result*, *they will either not tell you the truth or not welcome you at all to their household*.*” WBOT_4*

There was also a perception among WBOTs that PLWHIV are more likely to disclose to more senior WBOTs.

“*On more than one occasion*, *I was called whereby they could see that the patient didn’t want to disclose to them*. *So when I went*, *that person was a different person*, *he could relate more to me as an OTL [operational team leader]*.*” WBOT_2*

WBOTs perceived shared confidentiality among the multi-disciplinary team as a potential threat to confidentiality and accountability since the HIV status of PLWHIV is known to so many people in the facility: at least by a lay councillor, a nurse, a doctor, and the data entry clerk involved in the care of these patients.

“*The confidentiality becomes a challenge because now the patient will have disclosed to too many people and*, *when the information comes out*, *you never know who broke such confidentiality*.*” WBOT_1*

The structure of the household also has the potential for compromising confidentiality.

“*If we go into the house and you find that there is a two-room house we try looking for an alternative place; like you look for a place that will be at least far from everyone but inside the yard and then go there to talk to the person*.” *WBOT_1*

WBOTs were further concerned that HIV-related counselling and testing in the household could compromise confidentiality through the observation of non-verbal clues. They cited concerns about inadvertent disclosure by the persons being tested.

“*The reaction of the person gives the other family members a particular outcome because they already know what the person is doing; so*, *if the test comes positive and the person’s reaction is not good*, *then they conclude that the person is positive*.*” WBOT_4*

#### 4.2.3 Facility- and health system-related barriers

There are many different role players involved in the provision of HIV services. While the HIV/AIDS, sexually-transmitted infections and TB (HAST) programme involves WBOTs in meetings about PLWHIV, their involvement is limited to performing HIV testing and tracing people who have defaulted from HIV care.

Some PLWHIV supported the idea that WBOTs should only be involved in tracing patients who have defaulted treatment.

“*I think they must come*, *but not to those who are attending clinic regularly; but they must follow up those who don’t come (to the clinic)*.*” PLWHIV_3*

However, some WBOTs didn’t like the idea of only being involved once patients have become lost to follow up.

“*They don’t involve us*, *we never hear of HIV patients who are compliant on treatment*.*” WBOT_4*

In addition, WBOTs believe that facility staff do not prepare patients well enough for WBOT services.

“*They are not made to understand what WBOT is and what WBOT does and how are they going to assist them*.*” WBOT_3*

Community health workers refer people to the clinic on a regular basis, but there was a concern that some of these people are not well received at the clinics.

“*Two days back we referred a client*, *but they come back to us saying that the staff refused to take the referral*.” *WBOT_2*

Community health workers sometimes come across patients who do not have treatment, but are unable to assist them because of facility policies. An example given by one community health worker was of a patient who had relocated from another province without a referral letter.

“*So in the clinic they are only giving her weekly medication*, *so she still needs to get the letter so that she can be transferred to Gauteng*. *She is struggling to get the letter or proof of her medication*, *because KZN (KwaZulu Natal) is far from here and we haven’t gone back to her*.*” WBOT_3*

Neither PLWHIV nor WBOTs thought that HIV post-test counselling was done adequately in facilities. This placed a great burden on community health workers, as they have to re-do the counselling.

“*They aren’t doing it (post-test counselling)… They just told me everything will be well*, *you will be ok; they never sat down with me and do proper post-test counselling*. *They just asked me*, *are you here to test*? *I said yes*, *they pricked me*, *I never received counselling at all at the facility*.” *PLWHIV_1*

“*They are busy*, *she (lay counsellor) just said*, *after telling you what I am about to tell you*, *please don’t panic*. *Do you know about HIV*? *I responded and said*, *I have heard people talking about it*. *She said*, *you are HIV positive but don’t lose hope*. *They (lay counsellor) never sat down with me and counsel[led] me*. *I started getting information from [my] brother and sister (community health workers)*.*” PLWHIV_1*

WBOTs thought that an association existed between poor counselling and the testing targets that lay counsellors have to reach.

“*When they (lay counsellors) are testing people*, *they are thinking of targets*. *While you are busy taking long with counselling*, *others (lay counsellor) are busy testing more patients*. *They have to be fast*.*” WBOT_1*

“*If someone tested positive and they want to rush the process*, *that results in more frustration which patients have and what we come across in the community as community health workers*, *because those clients were rushed through the process*. *Rather than concentrating on the patients*, *the counsellors are concentrating on the targets*.*” WBOT_1*

“*They are busy with the numbers because they have allocated targets so that [they] can’t give more and appropriate counselling because they need to reach their target of counselled clients*.*” WBOT_4*

Community health workers reported that patients providing incorrect home addresses was one of the biggest barriers to HIV care in the community. Wrong or incomplete addresses significantly impaired community health workers’ ability to trace patients.

“*I have a list here which I received today*: *there are twenty people who we have to trace*: *fifteen of those are incomplete and only five have proper address*, *of which of those five addresses*, *we don’t know whether we will find the person or not*.*” WBOT_3*

The accuracy of this information was assessed in five randomly selected clinics, using tier.net, an electronic patient management system with modules to capture patient-level data on HIV-related counselling and testing, pre-ART and ART services, as the data source. There was great variability between the five clinics with the proportion of entries with no home address or no cell phone number recorded ranging from 2.9%– 55.6% and 0%– 37% respectively (Fig 1). These numbers do not include those with incorrect or incomplete addresses.

Protests from community health workers that they cannot trace patients without addresses seemingly fall on deaf ears.

“*I told him (linkage officer) there is no need for such a list with wrong and incomplete address as the results won’t have any effects on the list*, *but he insisted that we need to check these people*. *But we cannot let community health workers go around the inner township checking on these people because it will be just a waste of time*.*” WBOT_3*

Since community health workers’ time in the community is closely monitored by a team leader, they felt it is essential that time is not wasted by looking for incorrect addresses.

“*The issue of wrong address make us to spend a lot of time in one area and then we finally get the place and you find that*, *when we enter*, *it is the wrong address*: *there is no person whom we are looking for*, *…*.*so we end up spending a lot of time in one area rather than covering more people*.*” WBOT_3*

Various reasons were offered for people providing the clinic with incorrect addresses. The predominant reason offered by WBOTs was that people do not want to go to their nearest clinics and fear being turned away at other clinics if they are not from that area.

“*According to me*, these people are choosey because people from area A* come to clinic B* because the nurses in clinic A*, they are strict; and so they come to B* and they give wrong addresses so to receive services because, *if not*, *they will be asked why don’t you go to your local clinic*.” WBOT_3 (*area and clinic name anonymised to protect confidentiality)

Particular clinics are perceived to be lenient with regards to the requirement of patient identity documents. WBOTs reported that these clinics end up being dominated by foreign nationals who give incorrect addresses.

“*When you can go to the clinic*, *90% of the attendees you will find are foreign nationals*. *These foreign nationals*, *they don’t have identity documents or asylum*. When they go to clinic X*, *they don’t offer them services*, so when they come to [clinic] Y* they are attended to, so they end up giving wrong addresses and left the community health worker group in [clinic] Y* having clients to be traced, but they don’t reside in Y*.” WBOT_3 (*area and clinic name anonymised to protect confidentiality)

### 4.3 The perceived value of WBOTs and home-based HIV care

While it was not the intention of this study to question the effectiveness of the WBOT intervention, valuable information was offered by PLWHIV on the importance of this intervention.

“*This thing (WBOT intervention) motivates us*. *When they come to visit us*, *it gives us that support*, *they care*, *so we are motivated not to give up*. *It (home-based HIV care is) a good initiative*.*”PLWHIV_1*

“*They made me to be alright*. *First month I wasn’t ok; they taught me a lot*.*” PLWHIV_1*

PLWHIV currently receiving care from WBOTs reported that they only started understanding their illness when they were visited by WBOTs.

“*They came at the right time; I was asking myself what I was living for*. *When they came*, *they guided me*. *I wish God could give us more people like them*. *They know how to counsel people*, *they don’t harass you*. *When they came*, *I was very confused; when I see them coming in*, *I become very happy*.*” PLWHIV_1*

### 4.4 Recommendations by participants

Participants had several recommendations for dealing with perceived barriers to home-based HIV care.

WBOTs recommended that every clinic should have a WBOT office and that all newly-diagnosed persons should pass through this office on the day of treatment initiation. WBOTs believed that PLWHIV were most likely to trust them if they had met them at the facility level before.

“*I think they must really promote us*, *they must make people aware of the WBOT services especially during the testing and counselling*, *and even the community at large*, *because people are not aware of us and they don’t easily welcome us*, *but I believe that*, *if they were aware of WBOT*, *they would show a different attitude towards WBOT service providers*.*” WBOT_2*

In one of the clinics, WBOTs reported doing health talks in the clinic, including talks about the roles and functions of WBOTs. In this way, most patients came to know them and it became easier for them to conduct household visits.

“*Before they were not opening for us*, *but since we started doing health talks at the clinic and introducing the role of WBOTs*, *they now welcome us to their households*.*” WBOT_4*

“*They used to say*, *oh it is those ones for HIV*. *Now they understand that we aren’t just dealing with HIV*.*” WBOT_4*

There was a belief that WBOTs who were themselves HIV-infected, could perform home-based HIV care better as they would have a richer understanding of the situation of PLWHIV compared with those who were uninfected.

“*If they are also HIV positive*, *rather than (community health workers who are HIV-negative) just people who know nothing about what we have been through; someone going through the same experience won’t come to a household with an attitude*.*” PLWHIV_2*

Some WBOTs also indicated that they needed extra training on HIV-related counselling and testing.

“*We need training about HIV testing and counselling*, *because you find that you are at the household and a client wants to test and we can’t do that as we are not trained on that*.” *WBOT_2*

There was also a recommendation that HIV services should not focus on the individual, but rather have a comprehensive approach involving the whole family.

“*(To improve treatment adherence and support by family members)*, *there should be family (HIV) counselling*. *It will be good to focus on the family rather than a patient*. *So*, *the HIV care services should have a family focus rather than a patient focus*.*” WBOT_2*

Some PLWHIV suggested that WBOTs should focus mostly on men, as they are the ones who are least likely to attend the clinic.

“*Men are scared of the clinic*, *they don’t like coming to the clinic*. *These services should be directed to [at] them*.*” PLWHIV_2*

It was also recommended that PLWHIV should be trained as community health workers in order to take on roles of offering home-based HIV care.

“*They (*Department of Health*) should take us to go offer the services*, *whether we volunteer or be employed*, *because*, *even us here at the club*, *we are able to counsel and advise each other because of the experience we are having*.*” PLWHIV_3*

Linking home-based HIV care to existing structures, like adherence clubs, was recommended and this was seen to have the potential to improve the relationship between WBOTs and PLWHIV.

“*Before they (nurses) initiate any new clients they should first come to WBOT*, *as we are the ones who will continue with care in the community*, *currently it not like that they take them straight to the adherence club*.” WBOT_4

## 5 Discussion

This study which assessed the experience and value of, as well as the barriers to community health workers offering home-based HIV care and related services in Tshwane district yielded imported findings that are relevant to the current focus on Community-oriented Primary Care. While home-based HIV care was seen as a support strategy which could motivate patients to continue taking their medication, the unpredictability of patients’ responses to HIV test results, incorrect addresses, fear of stigma through association with WBOTs, little or no preparation of patients for home-based HIV care, and lack of confidentiality and trust were raised as potential barriers.

In addition, fears were expressed about the occupational health and safety of community health workers. While there are many guidelines that address this at facility level, there are limited resources addressing the safety of community health workers in the community. South Africa has the highest prevalence of TB/HIV coinfection in the world [16]. Part of offering home-based HIV care includes meeting people who have defaulted from treatment as well as those newly diagnosed with TB/HIV but who are not yet on treatment. This could place community health workers at risk since these individuals are potentially infectious, possibly also with drug-resistant organisms. The fact that some community health workers report having no masks to protect themselves from TB is therefore very concerning. Similar concerns were raised in a study [17] in which 39.1% (n = 270) of community health workers complained about inadequate protection from contracting TB. These fears are not unfounded: In a South African decade-long cohort study, health workers were reported to have a TB incidence of 1,496·32 per 100,000 compared to an incidence rate of 719·37 per 100,000 in the general population [18].

There is a concerted effort in South Africa to achieve the UNAIDS 90 90 90 targets [19]. Concerns were, however, expressed that the quality of HIV counselling was compromised by the pursuit of large numbers of HIV-infected patients tested and enrolled in treatment programmes, as required by donors. With lay counsellors seeing up to 25 patients a day on a busy day and also having many other duties apart from HIV counselling [20], the quality of counselling is easily compromised. While HIV post-test counselling is known to improve treatment adherence and virological suppression [21], WBOTs reported that many patients had not received adequate counselling in the facility. Even though this necessitated time and energy spent on re-counselling at the household level, the findings of this study suggest that this counselling does assist patients to better understand their illness. Formalizing a model of extending HIV post-test counselling through the involvement of WBOTs could be effective and serve as a support structure which could motivate patients to continue taking their medication [22].

WBOTs expressed the sentiment that their role should not be limited to only tracing defaulters, but should be expanded to support patients from the day they are diagnosed. This is supported by a systematic review [23] which reported on the positive outcomes achieved secondary to the expanded role of WBOTs in the management of HIV in terms of patient support (counselling, home‐based care, education, adherence support and livelihood support) and health services support (screening, referral and health service organization and surveillance) in sub-Saharan countries. These activities would fit nicely with the family focused home-based HIV care suggested by WBOTs in this study.

Many perceived barriers, which could undermine the important role of WBOTs, were raised. According to Katz *et al* [24], internal and external stigma are important barriers to treatment initiation. Similarly, results from our study suggest that fear of stigmatization is also a major barrier to home-based HIV care. Fear of the association that exists between WBOTs who do repeated household visits and serious illness was raised and such visits were reported to be attracting unwelcome attention. Just as in some studies where infrastructural challenges were reported to create unintended stigma, since specific buildings were exclusively used as ART clinics, it was reported in this study that WBOTs were seen as those offering just HIV care [25]. This fear of being associated with WBOTs was exacerbated by the symbols used by such teams, such as cars branded with signs of the clinic and the Department of Health and the wearing of standard medical uniforms. The discomfort of PLWHIV with WBOTs visiting them in uniforms was contrary to the belief expressed by some WBOTs in this study that uniforms assisted them in gaining access to a household. It is also contrary to the facility-based care perception that uniforms symbolise confidence, intelligence, trust, and safety [26]. In the community, wearing a uniform was seen as a symbol of someone who offers home-based HIV care. Further research into this aspect of care is critical.

Results of this study show that there is a degree of mistrust towards WBOTs since PLWHIV were uncertain whether they would keep their HIV status confidential. In fact, some cases of a breach of confidentially were reported. Similar to the findings of Grant *et al* [27] who reported that inadequate levels of confidentiality and trust were key barriers to the acceptability of community health workers delivering maternal and child health services, mistrust between PLWHIV and community health workers seemingly attenuate the acceptance of home-based HIV services offered by WBOTs. To improve confidentiality and trust, Grant *et al* [27] suggested that community health workers’ training on confidentiality and navigating complex relationships should be strengthened in conjunction with the provision of adequate supervision and support. Similarly, Hai *et al* [28] reported that training is an essential factor in practicing confidentiality.

The recruitment strategy for community health workers should also be reconsidered. While there are many logistical advantages of recruiting community health workers to work in their hometown, the results from this study suggest that this approach could be problematic for home-based HIV care. Other studies [27,29] reported the same challenges regarding the complexity of relationships and familiarity between community health workers and household members staying in the same community. It was suggested that community health worker training and support should focus more on how to navigate these complex relationships [27]. Furthermore, since health care professionals’ perceptions of community health workers’ competence and trustworthiness is fundamental to their credibility in the community, efforts to engender trust between personnel at health care facilities, community health workers and community members should be encouraged [30].

According to Chellaiyan *et al*, the quality of the infrastructure of facility-based HIV-associated counselling and testing is regarded as an essential component of the quality of counselling offered, with a separate and private counselling room regarded as essential [30]. This poses a significant challenge to home-based HIV care as each household’s infrastructure is unique, with no guarantee of confidentiality. From the results of this study, patients’ responses after receiving HIV results are unpredictable, with one community health worker reporting fearing for her physical safety after delivering the bad news. According to Naik [31], an impersonal setting, lack of compassion, and the unavailability of support staff may lead to violent reactions towards bad news. This insecurity can affect the performance of community health workers [32]. There is an urgent need for more research to be conducted on how to improve the physical safety of community health workers as they continue offering home-based HIV care.

Immigration policies and discrimination in health facilities have previously been reported as barriers to offering care to foreign nationals in South Africa [33]; home-based HIV care is no exception. A large number of patients reportedly didn’t have identity documents and therefore did not go to their nearest clinic but rather went to clinics where they could receive help without providing formal documentation. This however meant that they had to provide incorrect addresses so that they could be accepted in these clinics, thereby frustrating WBOT services. The need for an identity document as a prerequisite to access care should be revisited.

WBOTs recommended that every clinic should have a WBOT office in line with the “Ideal Clinic” recommendations [34]. It was also recommended that WBOTs should receive more training on HIV, that PLWHIV should be trained as community health workers, and that home-based HIV care should be linked to existing support structures. It is, however, important to note that the South African Department of Health is moving away from disease-specific community-based services towards a more comprehensive approach [35]. While employing people based on their disease status might seem to be at odds with this approach, a person living with HIV could add a valuable perspective as part of a WBOT.

This study has some strengths and limitations. One of the strengths is that the WBOTs who participated in the study were highly experienced with deep knowledge about home-based HIV care. While the sample size was small, participants were recruited from eleven clinics across Tshwane. One of the limitations of this study is that it was restricted to a relatively small area with a small number of participants and focus groups, which could have prevented the study from obtaining meaning saturation, especially for complex or subtle issues. Participants came mostly from an urban community and were dominated by female participants (81%) as can be expected from the gender proportions of PLWHIV in care as well as community health workers in the country [36]. This may have prevented identification of nuances influenced by demographic characteristics such as gender and geographic location [14].

## 6 Conclusion

To successfully implement effective home-based HIV care in South Africa, it is important to address perceived barriers to such care and involve all stakeholders, from decisionmakers to community health workers to PLWHIV. In addition, recommendations offered by people providing and receiving these services should be seriously considered. The most pertinent issues in need of attention are integrating WBOTs into clinics and existing support structures, improving training on confidentiality and HIV-related counselling and testing for community health workers, and rethinking the recruitment, scope of work and safety of WBOTs. Finally, the impact of the requirement for identity documents, and the acceptability and value of community health worker uniforms should be explored.

## Supporting information

S1 Table Interview guide. (DOCX)
