# Supplementary material for: “They come and knock at the gate until the neighbours see”. Perceived barriers and benefits of implementing HIV care at the community level in Tshwane district: A qualitative study
Source: PLoS One. 2020 Oct 15;15(10):e0240740. doi: 10.1371/journal.pone.0240740 (PMC7561185; doi:10.1371/journal.pone.0240740)
Supplement: S1 Table — (DOCX) [file pone.0240740.s001.docx]

# Interview guide

This interview interrogates three aspects of care 1) Experience of community health workers offering home-based HIV care or experience of people living with HIV with home-based HIV care ; 2) The perceived value of ward-based outreach teams (WBOT) and home-based HIV care; and 3) Barriers to conducting home visits. At the end of the interviews, a fourth area is explored where participants are given an opportunity to suggest recommendations to improve HIV care in the community.

| WBOT member | PLWHIV |
| --- | --- |
| 1. Experience of CHWs offering HBHIVC or experience of PLWHIV with HBHIVC; | |
| Please share your experience in working with PLWHIV | Have you ever been visited by WBOT members? |
| For how long have you been working with PLWHIV | Please share your experience in receiving care from WBOTM (door to door people) |
| Any other comment on experience. | Any other comment on experience |
| 2) Barriers to conducting home visits. | |
| What challenges have you faced in offering HBHIVC? | What challenges do you face or could you potentially face when visited by WBOTM? |
| Any other comments? | Any other comments? |
| 3) The perceived value of WBOTS and HBHIVC | |
| From your experience what is the value of offering HBHIVC? | What value do you think WBOT members bring or could bring to PLWHIV? |
| Any other comment? | Any other comment? |

^[[1]](#footnote-1)^HBHIVC – Home base HIV care ^[[2]](#footnote-2)^WBOT – ward based outreach team ^[[3]](#footnote-3)^PLWHIV – People living with HIV ^[[4]](#footnote-4)^WBOTM – ward based outreach team member

1. [↑](#footnote-ref-1)
2. [↑](#footnote-ref-2)
3. [↑](#footnote-ref-3)
4. [↑](#footnote-ref-4)
